# S81. NEUROCOGNITIVE FUNCTIONING IN SCHIZOPHRENIA AND BIPOLAR DISORDER DURING THE REMISSION AND THE PSYCHOTIC STATES

**DOI:** 10.1093/schbul/sby018.868

**Published:** 2018-04-01

**Authors:** Deniz Ceylan, Berna Binnur Akdede, Emre Bora, Ceren Hıdıroğlu, Zeliha Tunca, Köksal Alptekin, Ayşegül Özerdem

**Affiliations:** 1 Izmir University of Economics; 2 Dokuz Eylül University, School of Medicine

## Abstract

**Background:**

Previous literature comparing cognitive functioning between bipolar disorder (BD) and schizophrenia (Sch), particularly focused on remitted patients with BD (i.e. euthymics) and clinically stable patients with Sch; and suggested milder cognitive impairment in BD in comparison to Sch. Acute psychotic symptoms may lead poorer cognitive functioning in both disorders. Limited evidence suggests milder deficits in psychotic mania than in acute psychosis in Sch. We aimed to compare cognitive functioning in Sch and BD during the remission and the psychotic states.

**Methods:**

Several domains of cognitive functioning were compared among patients with BD who had a history of psychosis [32 with a current psychotic manic episode, 44 in euthymia for at least 6 months] and patients with Sch [41 with psychotic symptoms, 39 remitted according to Andreassen et al. criteria (2006)] in comparison to 55 healthy controls (HC). Participants performed a cognitive battery including Wisconsin Card Sorting, Rey Auditory Verbal Learning, Stroop, Auditory Consonant Trigram, Trail Making, Digit Span, Controlled Word Association, Category Fluency and Digit Symbol tests.

Principal components analyses were performed to extract the ‘global cognition’ factor and for dimensionality reduction to identify neurocognitive domains among patients with BD and Sch. The optimum number of cognitive components was identified by inspecting the scree plot. Each factor score was assessed for normality by calculating tests of skewness and kurtosis. The factor scores were compared between patients with Sch and patients with BD using two-way analyses of variance (ANCOVA) adjusting for age. Pairwise comparisons with Bonferroni corrections were used for post-hoc analysis.

**Results:**

Mean age, sex ratio levels of education were similar among patients with BD, patients with Sch and HCs. Principal components analyses revealed a global cognition factor that explains 52.6% of variance and a subsequent PCA revealed 5 factor domains including processing speed, verbal memory, visual memory, working memory and planning. Both patients with BD and patients with Sch have significantly poorer global cognition (p<0.001; p<0.001), processing speed (p<0.001; p<0.001); verbal memory (p<0.001; p=0.007); visual memory (p=0.033; p=0.016) and planning (p=0.011; p=0.006) than HCs. Patients with BD presented higher scores in global cognition, processing speed (p=0.010) and verbal memory (p=0.011) than patients with Sch (p<0.001). Global cognition and processing speed domains differ among groups with respect to both diagnosis [F=18.466, p<0.001; F=7.864, p=0.006] and state [F=8.910, p=0.001; F=3.958, p=0.048]. Processing speed, but not other components, displayed a significant interaction between diagnosis and state [F=14.808, p<0.001)].

**Discussion:**

Deficits in global cognition was milder in BD, than those in Sch in both the remission and the psychotic states. Although diagnosis seems to be the major factor affecting the cognitive performance, our data present significant interactions of diagnosis and state in processing speed.

